# Polyhydroxyalkanoate production in *Pseudomonas putida* from alkanoic acids of varying lengths

**DOI:** 10.1371/journal.pone.0284377

**Published:** 2023-07-20

**Authors:** W. Dirk Sikkema, Andrew J. Cal, Upul I. Hathwaik, William J. Orts, Charles C. Lee

**Affiliations:** Bioproducts Research Unit, USDA-ARS-WRRC, Albany, CA, United States of America; Karl-Franzens-Universitat Graz, AUSTRIA

## Abstract

Many studies have been conducted to produce microbial polyhydroxyalkanoates (PHA), a biopolymer, from *Pseudomonas* sp. fed with various alkanoic acids. Because this previous data was collected using methodologies that varied in critical aspects, such as culture media and size range of alkanoic acids, it has been difficult to compare the results for a thorough understanding of the relationship between feedstock and PHA production. Therefore, this study utilized consistent culture media with a wide range of alkanoic acids (C7-C14) to produce medium chain length PHAs. Three strains of *Pseudomonas putida* (NRRL B-14875, KT2440, and GN112) were used, and growth, cell dry weight, PHA titer, monomer distribution, and molecular weights were all examined. It was determined that although all the strains produced similar PHA titers using C7-C9 alkanoic acids, significant differences were observed with the use of longer chain feedstocks. Specifically, KT2440 and its derivative GN112 produced higher PHA titers compared to B-14875 when fed longer chain alkanoates. We also compared several analytical techniques for determining amounts of PHA and found they produced different results. In addition, the use of an internal standard had a higher risk of calculating inaccurate concentrations compared to an external standard. These observations highlight the importance of considering this aspect of analysis when evaluating different studies.

## Introduction

Much of modern society is dependent upon products manufactured from plastics. Most of these plastics are derived from “old” carbon, i.e., carbon fixed millions of years ago in petroleum or coal. These materials are often resistant to biodegradation, which initially was perceived to be advantageous; however, it has become evident that this lack of degradability is problematic. Even with extensive plastic recycling, significant amounts of plastic end up in landfills [1] or are simply discarded into the environment [2, 3]. Even when disposed plastic is incinerated, it is an environmental problem, since it will contribute to atmospheric CO_2_ as “old” carbon and can introduce toxins into the air. Another aspect of the plastic economy is that the manufacturing process itself can introduce environmental contaminants, e.g., vinyl chloride synthesis byproducts in the manufacture of PVC [4].

Polyhydroxyalkanoates (PHAs) represent a potentially more sustainable plastic although this aspect will be dependent on many factors, such as the environmental impact associated with the production of the fed carbon substrates. PHAs are produced by many bacteria as a carbon storage molecule, similar to starch or glycogen [5–7], often when carbon is in excess and other nutrients (especially nitrogen or phosphorus) are limiting [8, 9]. Medium chain length (mcl)-PHAs are composed of 3-hydroxy fatty acids of 6 to 12 carbons in length. They were first observed when De Smet et al. reported that *Pseudomonas oleovorans*, when grown on octane, produced intracellular inclusions [10]. Later, it was shown that *P*. *oleovorans* would synthesize mcl-PHAs and that the composition of the mcl-PHA was in large part reflective of the carbon source upon which the bacteria was grown, e.g., when grown on octane or octanoic acid, the primary monomer in the mcl-PHA produced is 3-hydroxyoctanoic acid [11–15].

Subsequent work has shown that a significant number of *Pseudomonas* species can produce mcl-PHAs. Haywood et al. showed that *P*. *aeruginosa*, *P*. *oleovorans*, *P*. *putida*, and *P*. *fluorescens* could grow on C6 (hexanoic) through C10 (decanoic) alkanoic acids and produce mcl-PHAs reflective of the respective alkanoic acid [16]. Timm and Steinbuchel examined PHA production from a large number of *Pseudomonas* species grown on either gluconate or octanoic acid and found that *P*. *aeuroginosa*, *P*. *aureofaciens*, *P*. *citronellolis*, *P*. *chlororaphis*, *P*. *denitrificans*, *P*. *marginalis*, *P*. *mendocina*, *P*. *oleovorans*, *P*. *putida*, *and P*. *syringae* were able to produce mcl-PHAs from both [17].

Others have also shown that mcl-PHAs can be produced from various alkanoic acids including: oleic [18, 19], octanoate and oleic [20], 10-undecenoate and nonanoate [21], octanoate [22–25], laurate, myristate and oleate [26], octanoate, nonanoate, undecanoate and oleic [27], decanoate [28], oleate [29], nonanoate [30], C3, C5, C7 and C9 alkanoates [31], oleic acid [32, 33], C6 through C9 alkanoates [34], oleic, erucic (C22 monounsaturated), and nervonic (C24 monounsaturated) [35], octanoate, laurate, palmitate and oleate [36], C6, C8, C10 and C12 alkanoates [37], waste free fatty acids [38], and C6, C8, C9, C10, C12 and C14 alkanoates [39, 40].

The above studies were conducted in multiple laboratories using various growth conditions. For instance, different types and amounts of feedstocks were used in assorted media formulations. In addition, various *Pseudomonas* strains were used in the experiments. Furthermore, different methods of PHA quantification were employed in these studies. These variations create difficulties in developing a clear understanding of the relationship between the feedstock and PHA production. In this study, we analyzed the microbial PHA produced from three *Pseudomonas* strains when a wide range of alkanoic fatty acids (C7-C14) was used as feedstock at the same total carbon concentration loading. We were interested in determining how the use of these different feedstocks would impact PHA production among the different strains.

## Materials and methods

### Chemicals, bacterial strains, and growth conditions

All chemicals and reagents were obtained from Sigma-Aldrich (MO, USA) unless otherwise specified. *Pseudomonas putida* B-14875 was obtained from the ARS Culture Collection (NRRL, USDA-NCAUR, IL, USA). *P*. *putida* KT2440 was obtained from W. Pat Wechter (USDA-ARS, SC, USA). *P*. *putida* GN112 was generously provided by Dr. Josef Altenbuchner (Universitat Stuttgart, Stuttgart, Germany). *P*. *putida* GN112 was originally derived from *P*. *putida* KT2440 and adapted for markerless deletion. The strain contains deletions of a 26,049 bp region related to surface adhesion proteins (lapA) and a 64,722 bp deletion related to flagellum biosynthesis [41]. For long term maintenance, glycerol stocks were prepared from cells grown in LB (1% NaCl, 1% tryptone, 0.5% yeast extract), made to 20% with glycerol, frozen, and stored in a -80°C freezer. Working stocks were prepared from the glycerol stocks and maintained on LB plus 1.5% agar plates with colony transfers every two to four weeks. Inocula for shake flask cultures were prepared by inoculating 20 mL of LB plus 20 mM of sodium octanoate in a sterile 125 mL flask with 1–3 colonies from an LB plate and shaking at 200 rpm overnight at 30°C. Cells were grown in 1 L baffled shake flasks with 200 mL of media containing a fatty acid carbon source (as the sodium salt), 20 mM ammonium sulfate, 20 mM potassium phosphate buffer (pH 7.5), 50 mM Tris-HCl (pH 7.5), 0.8 mM magnesium sulfate, 140 μM calcium chloride, 2 μM copper sulfate, 0.41 μM sodium molybdate, 50 μL of a 3.8% solution of ferric EDTA (MP Biomedicals, CA, USA), and 1.0 mL of a trace mineral solution [42]. The fatty acid carbon sources were obtained from Sigma-Aldrich (hexanoic thru decanoic acid) or TCI America (undecanoic thru palmitic acid) (OR, USA). Fatty acids were converted to sodium salts by the addition of an appropriate amount of sodium hydroxide and then the pH was adjusted to 7.5. The concentration of fatty acid used was adjusted so that all cultures had approximately the same carbon content. The carbon source and the phosphate buffer were autoclaved separately and added to the media just before use. The ferric EDTA solution was filter sterilized and added prior to use. E* mineral salts media was prepared as previously described [14, 27]. Nitrogen and phosphate concentrations were adjusted so as to facilitate comparison to the media described above.

Cultures were inoculated with 4 mL of the overnight culture from above with a starting OD_600_ of between 0.08 and 0.1. Growth was monitored by determining the OD_600_ at 24, 48, and 68 hours. At 68 hours, the final pH was determined, and the cells were harvested by centrifugation at 5100 x g for 25 min. Cells were resuspended in water and recentrifuged, then resuspended in 50 mL of water in a 50 mL Falcon tube and pelleted at 11000 x g. The supernatant was poured off, and pellets were then frozen and lyophilized for 48h. Residual moisture from lyophilized cells was less than 1% as determined on a moisture analyzer (MB25; OHAUS, NJ, USA). Freeze-dried pellets were ground to a fine powder with a mortar and pestle for subsequent analysis.

### Measurement of PHA by GC-MS (gas chromatography-mass spectrometry)

Polyhydroxyalkanoates produced under various conditions were analyzed by gas-chromatography essentially as previously described [14, 17]. Approximately 10–15 mg of lyophilized cells (weighed to the nearest 0.01 mg) were suspended in 1.0 mL of chloroform in a 13 x 100 mm screw cap tube. One mL of methanol containing 15% (v/v) concentrated sulfuric acid was added. The tubes were sealed with a PTFE (polytetrafluoroethylene) lined cap and heated at 100–105°C for 120 min with intermittent shaking. After cooling, 1.5 mL of dH_2_O was added, and the tubes were vortexed for 15–20 sec. After separation, the organic (bottom) layer was transferred to a microcentrifuge tube containing approximately 50 mg of anhydrous magnesium sulfate, sealed, shaken and then centrifuged at top speed in a microcentrifuge for 5 min. The supernatant was then diluted into the autosampler vials (1:500) containing 1.0 mL of chloroform. Analysis of samples was conducted on a ThermoScientific Trace 1310 gas chromatograph (Thermo Fisher, MA, USA) coupled to a 155-place autosampler. The GC was equipped with a 30 m, ThermoScientific TG-5 MS column (25 mm ID with a film thickness of 0.25 μm). The GC was operated in the splitless mode with helium as the carrier gas at 2.0 mL/min. The injector temperature was 250°C. The oven was programmed with a 2 min hold at 40°C, followed by a temperature increase of 20°C/min to 240°C and a 4 min hold at 240°C. One μL was injected onto the column. Detection was accomplished using the coupled ThermoScientific IQS LT Single Quadrupole Mass Spectrometer. The mass transfer line was operated at 250°C, and the ion source temperature was set at 300°C. The mass spectrometer was operated to detect masses between 50 and 350 amu. Quantification and peak identification was obtained using the ThermoScientific Chromeleon software. Monomers in the PHA were identified on the basis of retention time and mass spectra (mass spectra being compared to a library of mass spectra). Quantification of PHAs in cells was accomplished using methanol-precipitated PHAs as a standard.

Both an external and an internal standard were employed. For the external standard method, material that was chloroform extracted and methanol precipitated was used as the standard. The purified standard for each alkanoate-grown culture was prepared from chloroform extracted, methanol precipitated material from the corresponding culture, e.g. chloroform extracted, methanol precipitated PHA from heptanoate-grown cells was used as the standard for heptanoate-grown cells. A standard curve was developed using five different concentrations of the material and three different PHA preparations. The sum of the areas for the methyl-3-hydroxyalkanoate was then plotted against the amount of purified PHA to develop the standard curve. Samples were run so as to have the sum of the methyl-3-hydroxyalkanoate from the sample fall midway along the standard curve. PHA amount was then calculated from the inverse of the slope of the line of the standard curve.

For the internal standard method, the area and amount of the standard (benzoic acid) was used with the sum of the areas from methyl-3-hydroxy alkanoates from known amounts of purified PHAs to develop a response factor. The average response factor was then used to calculate the amount of PHA in the unknown.

### Purification and gravimetric determination of PHAs

Purification of PHAs was accomplished by weighing about 500 mg of lyophilized cells (weighed to the nearest 0.01 mg) into a 15 mL glass vial. Ten mL of chloroform were added and the vials were sealed with PTFE-lined caps (#93000–928; VWR, PA, USA). The vials were heated at 75°C for at least 2 hrs and then cooled. The extract was then filtered through a glass fiber filter (25 mm, Sigma Z268364). The vial and extracted cells were then washed with about 5 mL of chloroform, and the entire filtrate was transferred to a pre-weighed aluminum pan. The filtrate was evaporated to dryness under a stream of air overnight. The pan was then weighed and the dried extract redissolved in 2–3 mL of chloroform. This material and washings were then precipitated in ice cold methanol (about 50 mL) and the precipitate was allowed to settle overnight in the cold. After precipitation, the pure PHA was then redissolved in a small volume of chloroform and transferred to a pre-weighed vial. The chloroform was then evaporated overnight, and the vials were weighed. The amount of material left in the vial was the gravimetric amount of PHA. This material was used for PHA standards in the GC/MS determinations as well as for the GPC analysis.

### Gel permeation chromatography (GPC)

GPC was accomplished by dissolving purified PHA in tetrahydrofuran at an approximate concentration of 10 mg/mL. Chromatography was performed by injecting 100 μL onto a Hewlett–Packard 1100 series HPLC (1.0 mL/min flowrate, THF continuous phase, 35°C) with two Agilent PL gel 10 μm Mixed-B columns in series coupled to multi-angle laser light scattering (DAWN Heleos-II, Wyatt Technology, Santa Barbara, CA) and refractive index (Agilent 1260 Infinity, dn/dc = 0.129) detectors. The mobile phase was tetrahydrofuran pumped at 1.0 mL/min at 37°C. The instrument was calibrated using low-polydispersity polystyrene standards.

### Statistical analysis

Statistical analysis was performed in the R environment [43] with the *agricolae* package [44] using the TukeyHSD function, and p-values have been adjusted for multiple comparison. Plots were constructed using the *ggplot2* package [45].

## Results and discussion

### Development of media

Previous demonstrations of polyhydroxyalkanoate (PHA) production from *Pseudomonas* sp. fed with alkanoic acids have utilized different standard media formulations, such as E* (Foster et al., 2005). These different conditions make it difficult to compare the results from these various studies. Thus, we chose to use a well-defined media formulation for all the experiments in this study. Our media contained 40 mM ammonium ion, 20 mM potassium phosphate, and 50 mM Tris-HCl, and the pH was adjusted to 7.5.

Table 1 compares the results of this study when *P*. *putida* B-14875, KT2440, and GN112 were grown on octanoate with other reports of *P*. *putida* grown on octanoate as well as the closely related *P*. *oleovorans*. The results show that the defined salt media (DS) described in this work approximately doubled the cell dry weight (CDW) titer of that described in most other reports. In addition, the percent of PHA was generally higher with the DS media compared to previously used formulations as seen in Table 1 although it is difficult to compare the data since various amounts of feedstocks were used across all the studies. The distribution of monomers was similar across all the media with the vast majority being hydroxyoctanoate. Having established the functionality of the DS media, this formulation was used for the remainder of the studies.

**Table 1:** Comparison of different media from literature with the present study.

Reference	Strain	Media	Carbon	Carbon	Carbon	NH_4_	PO_4_	CDW/L	%PHA	C5	C6	C8	C10	C12
			Source	(mM)	(g/L)	(mM)	(mM)	(g/L)						
[14]	*P*. *oleo* A 29347	Shwartz	oct	20.0	1.9	16.7	68.8	1.2	27.0		9.6	86.1	4.3	
[16]	*P*. *putida*	Shwartz	oct	34.7	3.3	150.0	104.0	NR	15.0		7.0	84.0		
[22]	*P*. *oleo* A 29347	Shwartz	oct	34.7	3.3	150.0	104.0	NR	11.0		5.0	92.0	3.0	
[46]	*P*. *putida* KT2442	E*	oct	20.0	1.9	16.7	68.8	1.0	39.0		6.3	92.0	1.7	
[19]	*P*. *putida* KT2442	E2	oct	10.0	1.0	8.4	64.4	NR	22.3		6.0	92.0	2.0	
[15]	*P*. *oleo* A 29347	E*	oct	10.0	1.0	16.7	68.8	1.5	41.0	1.0	6.0	75.0	17.0	
[24]	*P*. *oleovorans*	E*	oct	20.0	1.9	16.7	68.8	2.3	15.7		11.2	84.5	4.3	
[34]	*P*. *putida* KT2440	LB/Oct	oct	69.4	6.7	NR	NR	4.6	28.9		13.4	81.0	5.6	
[36]	*P*. *putida* Bet01	salts	oct	10.0	0.8	16.7	76.6	9.8	49.7		8.1	76.2	11.0	4.7
[22]	*P*. *oleo* A 29347	E	oct			7.6	46.5		28.0			99.0		
[40]	*P*. *putida* LS46	RMM	oct	20.0	1.9	30.2	19.9	2.4	56.2		6.5	88.5	3.8	1.1
this study	*P*. *putida* GN112	E*	oct	25.0	2.4	16.7	68.8	2.06						
this study	*P*. *putida* GN112	DS	oct	25.0	2.4	40.0	20.0	6.7						
this study	*P*. *putida* B-14875	DS	oct	75.0	7.2	40.0	20.0	5.6	34.1		8.3	88.5	3.2	
this study	*P*. *putida* KT2440	DS	oct	75.0	7.2	40.0	20.0	5.6	30.2		7.0	89.8	3.2	
this study	*P*. *putida* GN112	DS	oct	75.0	7.2	40.0	20.0	6.2	33.8		7.5	91.0	1.5	

*P*. *oleovorans* ATCC 29347 included because *P*. *oleovorans* and *P*. *putida* are closely related. “CDW” corresponds to the cell dry weight after lyophilization of the collected cells. C5 through C12 indicates the size of methyl-3-hydroxy alkanoate produced in methanolysis, and the data reflect the relative percentage of each species. NR, not reported.

### Growth of *P*. *putida* strains B-14875, KT2440, and GN112

We tested the growth and PHA production of *P*. *putida* fed with sodium alkanoates ranging from C7-C14. In our experiments, we adjusted the concentration of the alkanoate supplied such that the carbon content of the media was the same for each alkanoate (2.4 g/L carbon per flask initially followed by additions of 2.4 g/L carbon at 24 h and 48 h (7.2 g/L total carbon)). This allowed us to make direct comparisons of the CDW titer and percent PHA for each alkanoate supplied. While the sodium salts of heptanoic through nonanoic acid were completely soluble at the initial concentration and pH (7.5 to 7.6), the higher carbon number sodium alkanoates (decanoic through myristic) were largely suspensions. However, all three strains exhibited little difficulty in utilizing the suspended alkanoates. This was indicated by the observation that when the OD_600_ determinations were made, centrifugation of the sample would produce no upper layer of alkanoate whereas media without cells would.

When the growth on all the substrates collectively was analyzed, there were significant differences in final OD between all strains (GN112 > KT2440 > B-14875) (Table 2). GN112 exhibited significantly higher ODs than KT2440 (p = 0.004) and trended toward higher CDW (p = 0.07). The final OD_600_ and the CDW for each strain yielded 1.0 OD_600_ equaling 0.42, 0.45 and 0.42 mg/mL CDW for B-14875, KT2440, and GN112, respectively. The final PHA titer (g/L) was significantly greater from GN112 compared to the other strains.

**Table 2:** Comparison of strains across all substrates.

Strain	68 hr	CDW	%PHA	%PHA	PHA titer
	OD_600_	(g/L)	(GC-ext)	(GC-int)	(g/L)
B-14875	12.2^a^ (2.4)	4.93 ^a^ (0.80)	28.5% ^a^ (7.9)	32% ^a^ (7.2)	1.44 ^a^ (0.53)
KT2440	13.9^b^ (2.3)	5.97 ^b^ (0.47)	34% ^b^ (7.1)	33.5% ^ab^ (5.2)	2.05 ^b^ (0.53)
GN112	15.7^c^ (1.8)	6.25 ^b^ (0.59)	34.5% ^b^ (7.1)	36% ^b^ (6.0)	2.18 ^c^ (0.54)

“CDW” corresponds to the cell dry weight after lyophilization of the collected cells. “(GC-Ext)” equals the percent PHA determined from the methanolysis of whole cells using purified PHA from a similar carbon source as an external standard. “(GC-Int)” equals the percent PHA determined from the methanolysis of whole cells using PHA from a similar carbon source as the standard relative to methyl benzoate as the internal standard. Standard deviations are shown in parentheses. Superscripts (a,b,c) denote TukeyHSD grouping, where means between groups have p < 0.05 after multiple test correction.

KT2440 is a strain that has been widely used in experiments to produce mcl-PHA, and GN112 is derived from KT2440. That GN112 performed better than KT2440 could be explained by the fact that GN112 has had approximately 1.6% of its genome (90771/5800000) deleted [41]. B-14875 is a strain in the NRRL culture collection that was obtained from municipal sewage in Indiana and has recently been reported to produce mcl-PHA from a glycerol rich byproduct [47]. The difference between KT2440 and GN112 compared to B-14875 might be explained by the fact that KT2440 was selected on the basis of hydrocarbon utilization whereas B-14875 was for its ability to degrade the insecticide carbaryl (NRRL strain description).

As shown in Table 3, strain B-14875 produced CDW of between 4.1 g/L and 6.1 g/L. In the same fashion, from Table 4, the titer range for strain KT2440 was between 5.6 g/L to 6.5 g/L. Similarly, from Table 5, strain GN112 yielded a CDW range of 5.5 g/L to 6.9 g/L.

**Table 3:** Growth and PHA production of *P*. *putida* B-14875.

Carbon		24 hrs	48 hrs	68 hrs	pH @	CDW	%PHA	%PHA	%PHA	%PHA
**Source**		**OD**_**600**_	**OD**_**600**_	**OD**_**600**_	**68 hrs**	**(g/L)**	**(GC-Ext)**	**(GC-Int)**	**Init**	**MeOH**
**Hep**	**Avg**	3.99	7.96	11.45	7.81	4.97	23.47	30.04	44.61	12.63
	**SD**	0.20	1.09	2.35	0.30	0.42	10.78	9.98	8.97	6.07
**Oct**	**Avg**	5.24	9.01	12.47	7.77	5.56	33.22	34.10	46.86	19.09
	**SD**	0.28	1.17	1.18	0.16	0.35	6.83	4.92	8.28	3.72
**Non**	**Avg**	4.31	7.00	12.92	7.18	4.05	28.78	33.35	40.20	28.36
	**SD**	0.47	1.52	3.81	0.52	0.37	10.71	7.83	4.77	4.87
**Dec**	**Avg**	3.55	6.39	10.75	6.83	4.43	27.30	31.32	36.74	26.79
	**SD**	1.34	0.46	1.17	0.30	0.56	4.10	4.43	2.96	2.10
**UnD**	**Avg**	4.96	7.67	11.62	6.64	4.72	26.41	38.49	37.98	20.70
	**SD**	0.17	0.22	3.10	0.38	0.99	4.99	5.78	3.28	0.49
**Lau**	**Avg**	5.11	8.91	10.65	6.84	4.56	32.17	30.19	44.98	30.78
	**SD**	0.27	0.81	2.24	0.34	0.67	4.64	3.72	5.87	4.06
**TrD**	**Avg**	6.14	10.60	14.18	7.60	6.12	34.64	35.25	44.06	21.99
	**SD**	0.35	0.73	1.22	1.17	0.28	8.05	6.60	5.42	5.11
**Myr**	**Avg**	2.89	7.81	13.87	7.11	5.07	22.66	22.41	29.03	21.05
	**SD**	0.05	0.50	1.33	0.22	0.24	4.07	3.94	5.74	4.50

“Hep”, “Oct”, “Non”, “Dec”, “UnD”, “Lau”, “TrD”, “Myr” are heptanoate, octanoate, nonanoate, decanoate, undecanoate, laurate, tridecanoate, and myristate, respectively, and correspond to the sodium salt of the alkanoic acid fed to each culture. Each number of “Avg” represents the average of six flasks. “SD” represents the standard deviation of the average above. “OD_600_” is the optical density of the culture at the time indicated. “CDW” corresponds to the cell dry weight after lyophilization of the collected cells. “(GC-Ext)” equals the percent PHA determined from the methanolysis of whole cells using purified PHA from a similar carbon source as an external standard. “(GC-Int)” equals the percent PHA determined from the methanolysis of whole cells using PHA from a similar carbon source as the standard relative to methyl benzoate as the internal standard. “Init” equals the initial percent mass of chloroform extracted material from freeze dried cells. “MeOH” equals the percent mass of the chloroform extracted, methanol precipitated material.

**Table 4:** Growth and PHA production of *P*. *putida* KT2440.

Carbon		24 hrs	48 hrs	68 hrs	pH @	CDW	%PHA	%PHA	%PHA	%PHA
**Source**		**OD**_**600**_	**OD**_**600**_	**OD**_**600**_	**68 hrs**	**(g/L)**	**(GC-Ext)**	**(GC-Int)**	**Init**	**MeOH**
**Hep**	**Avg**	4.14	8.40	13.83	7.68	5.75	32.96	37.39	38.06	22.33
	**SD**	1.73	2.53	1.92	0.18	0.40	5.57	4.97	1.46	5.06
**Oct**	**Avg**	4.14	8.40	13.83	7.70	5.65	30.14	35.77	40.01	19.56
	**SD**	1.73	2.53	1.92	0.21	0.36	6.66	5.56	3.70	4.94
**Non**	**Avg**	4.60	8.26	13.01	7.29	5.67	28.75	33.40	43.28	26.36
	**SD**	1.16	2.46	0.54	0.25	0.23	7.63	5.11	4.69	2.42
**Dec**	**Avg**	4.66	7.93	13.42	7.29	5.69	38.90	32.59	42.56	32.68
	**SD**	0.97	1.61	1.03	0.17	0.38	3.59	2.92	2.07	2.77
**UnD**	**Avg**	5.14	9.44	14.27	7.18	5.93	30.10	37.15	44.22	34.29
	**SD**	0.49	0.56	1.50	0.20	0.52	3.84	2.77	2.12	1.34
**Lau**	**Avg**	5.79	9.96	15.16	6.92	6.29	33.86	33.36	42.71	32.07
	**SD**	0.64	0.63	0.56	0.14	0.17	2.16	1.79	1.21	2.21
**TrD**	**Avg**	5.10	8.40	10.20	6.94	6.34	32.93	34.25	39.68	22.57
	**SD**	0.73	0.68	0.61	0.17	0.20	2.01	1.85	0.97	4.28
**Myr**	**Avg**	6.08	12.14	17.73	7.18	6.48	45.94	25.59	50.34	35.93
	**SD**	0.79	1.66	1.66	0.23	0.54	5.13	3.87	1.40	3.13

Abbreviations correspond to those indicated in Table 3.

**Table 5:** Growth and PHA production of *P*. *putida* GN112.

Carbon		24 hrs	48 hrs	68 hrs	pH @	CDW	%PHA	%PHA	%PHA	%PHA
**Source**		**OD**_**600**_	**OD**_**600**_	**OD**_**600**_	**68 hrs**	**(g/L)**	**(GC-Ext)**	**(GC-Int)**	**Init**	**MeOH**
**Hep**	**Avg**	4.36	8.98	13.86	7.92	5.68	33.46	35.39	40.29	26.74
	**SD**	1.20	1.92	2.25	0.24	0.44	11.01	9.43	8.75	8.08
**Oct**	**Avg**	5.02	9.80	15.23	7.73	6.19	29.92	33.74	40.66	20.80
	**SD**	0.41	1.08	1.58	0.23	0.47	6.11	4.62	3.53	6.00
**Non**	**Avg**	6.12	11.12	13.43	7.48	5.46	28.41	32.17	40.49	25.89
	**SD**	0.94	2.09	1.28	0.23	0.18	7.69	5.64	5.75	3.26
**Dec**	**Avg**	6.29	11.32	16.25	7.61	5.80	40.19	44.59	49.51	36.53
	**SD**	0.43	1.03	1.15	0.27	0.25	5.86	4.00	3.74	4.45
**UnD**	**Avg**	5.70	11.20	16.40	7.57	6.79	36.03	36.36	42.59	33.90
	**SD**	0.62	1.04	0.99	0.25	0.13	2.31	1.94	1.73	2.31
**Lau**	**Avg**	6.45	11.55	17.18	7.50	6.81	40.91	40.24	44.06	35.89
	**SD**	0.73	0.58	0.68	0.27	0.21	5.93	2.89	2.35	2.50
**TrD**	**Avg**	6.86	12.04	17.57	7.06	6.89	35.85	34.80	43.42	28.05
	**SD**	0.80	0.81	0.78	0.23	0.22	0.61	1.33	1.46	5.54
**Myr**	**Avg**	4.32	10.05	15.73	6.74	6.44	31.70	31.59	34.21	29.39
	**SD**	1.03	0.95	0.80	0.11	0.17	3.22	2.66	2.21	2.43

Abbreviations correspond to those indicated in Table 3.

**Fig 1:**
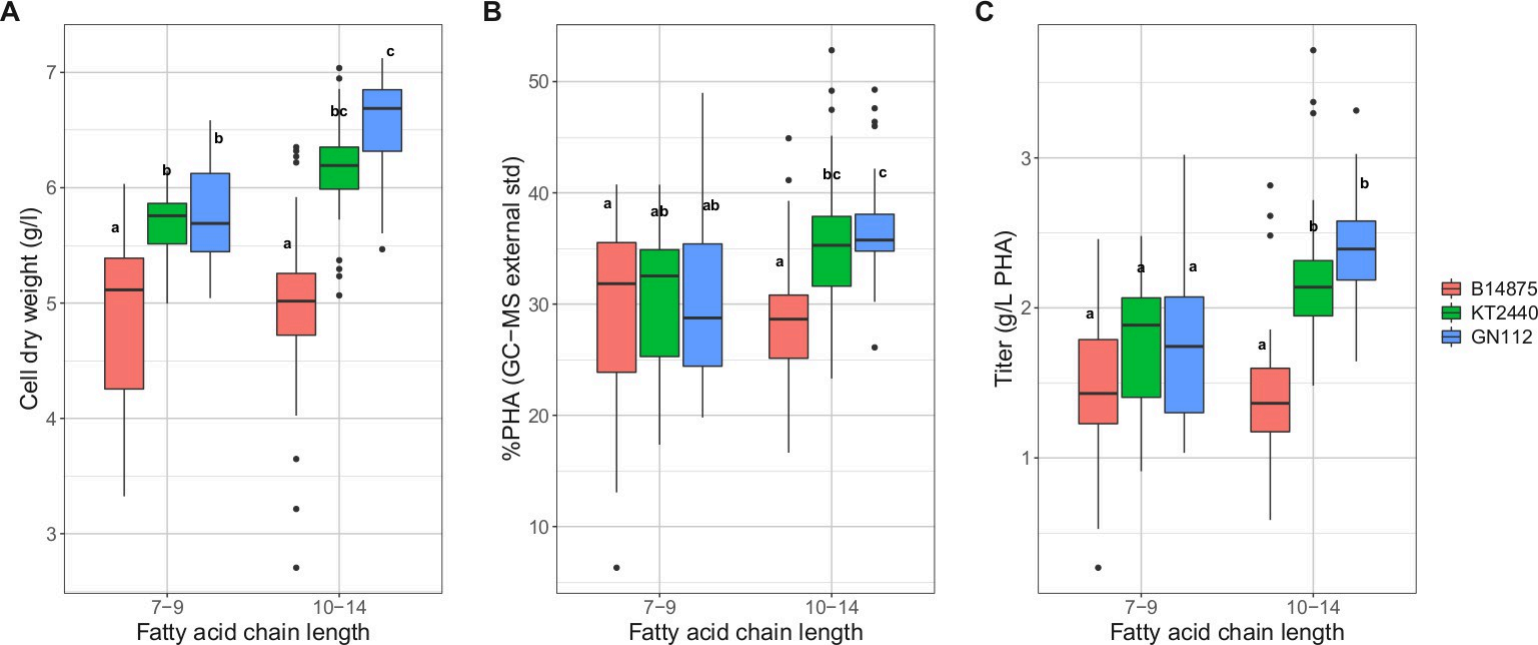
Comparison of three *P*. *putida* strains grown on substrates with different chain lengths. Effects of different alkanoic acid chain length feedstocks on (A) cell dry weight, (B) %PHA, and (C) titer. 7–9, alkanoic acid chain lengths C7-C9; 10–14, alkanoic acid chain lengths C10-C14. Grouping designations (a,b,c) denote TukeyHSD grouping, where means between groups have p < 0.05 after multiple test correction.

When the effects of using alkanoic acid feedstocks of varying chain lengths were analyzed, significant differences were observed among the strains (Fig 1). When the C7-C9 substrates were used, there was greater CDW from KT2440 and its deletion derivative, GN112, relative to B-14875 (Fig 1A). When the longer C10-C14 substrates were used, the CDW of B-14875 did not change but those for KT2440 and GN112 increased. With regards to %PHA, when C7-C9 substrates were used, all three strains had similar levels (Fig 1B). KT2440 and GN112 had increased %PHA with the larger C10-C14 substrates while B14875 remained unchanged. The final PHA production showed that all three strains had similar titers when C7-C9 substrates were used (Fig 1C). When C10-C14 feedstocks were used, the titers of both strains KT2440 and GN112 increased significantly compared to B-14875. GN112 also appeared to have a slightly higher titer that its parent strain, KT2440. Because the GN112 strain has significant genomic deletions, the lower metabolic load may be the cause of the increased PHA titer. The higher titers from KT2440 and GN112 when using the higher molecular weight alkanoates might be explained by the fact that these feedstocks have a greater proportion of reduced carbon. In contrast, B-14875 did not show any performance differences regardless of the alkanoic acid chain length. These general trends are supported by the ratio of carbon that was incorporated in the polymer to the total input carbon that was introduced as substrate (Table 6). The reason that B14875 and KT2440/GN112 have different responses to longer chain alkanoates could be due to potential variations in the strains’ fatty acid transporters or enzymes in the β-oxidation pathway [48–50].

**Table 6:** Mole percent of 3-hydroxy fatty acid in PHA present in whole cells.

Carbon												
**Source**	**Strain**											
		**C5**	**C6**	**C7**	**C8**	**C9**	**C10**	**C11**	**C12**	**C13**	**C14**	**(polymer/input)**
												**C**
**Hep**	**B-14875**	2.79		92.74		4.47						0.11
	**KT2440**	tr		98.27		1.73						0.18
	**GN112**	tr		98.70		1.30						0.18
**Oct**	**B-14875**		8.34		88.47		3.19					0.18
	**KT2440**		7.04		89.78		3.18					0.17
	**GN112**		7.47		90.95		1.57					0.18
**Non**	**B-14875**	2.06		28.76		68.80		0.39				0.12
	**KT2440**	1.43		25.20		73.18		0.18				0.16
	**GN112**	2.01		29.40		68.40		0.19				0.15
**Dec**	**B-14875**		6.77		55.87		37.36					0.12
	**KT2440**		5.75		50.11		44.14					0.22
	**GN112**		6.47		54.66		38.87					0.23
**UnD**	**B-14875**	2.54		21.53		54.63		21.31				0.13
	**KT2440**	1.80		19.57		50.81		27.82				0.18
	**GN112**	1.97		21.53		51.12		25.37				0.25
**Lau**	**B-14875**		5.54		45.16		35.43		13.87			0.15
	**KT2440**		5.78		45.22		35.68		13.33			0.21
	**GN112**		6.13		52.33		32.47		9.06			0.28
**TrD**	**B-14875**	1.99		18.49		50.97		22.43		6.11		0.21
	**KT2440**	1.87		18.03		50.98		22.83		6.30		0.21
	**GN112**	1.87		18.03		50.98		22.83		6.30		0.25
**Myr**	**B-14875**		6.41		51.45		33.04		8.07		1.04	0.12
	**KT2440**		5.95		48.05		35.28		9.53		1.19	0.30
	**GN112**		6.81		51.93		32.05		8.28		0.93	0.20

Abbreviations correspond to those indicated in Table 3. C5 through C14 indicates the size of methyl-3-hydroxy alkanoate produced in methanolysis. (polymer/input) C is the ratio of carbon in the polymer to the carbon of the total input alkanoate substrate fed to the culture. tr, trace.

### PHA production of *P*. *putida* strains B-14875, KT2440, and GN112 determined with different methodologies

Many studies that determine the amount of PHA produced in microorganisms use one of two common protocols: GC (coupled to a detector, such as MS) or gravimetric method. The GC method requires specialized equipment and more laborious sample pretreatment but does provide information on specific monomer composition. In contrast, the gravimetric method is much simpler and does not require any specialized equipment although it provides no compositional information. To better understand potential differences between these two methods in determining total titer of polymer, both were used to analyze all of our samples.

For the GC/MS method, an additional variable was tested: the use of either an external or an internal standard. We wanted to compare the external standard method versus the internal standard method because we observed that the response factors calculated for the internal standard varied significantly depending on the amount of PHA sample used in the determinations (S1 File). Since the formula used to calculate the PHA present in the unknown is:

Amount PHA = (Amount-Internal Standard x Area Unknown PHA x Response Factor)/ Area-Internal Standard when the response factor is high, an artificially higher amount of PHA is calculated. To circumvent this problem as much as possible, the unknown amount was weighed to produce PHA approximately between the highest and lowest amount of PHA internal standard. Overall, we found that the external standard method was more accurate over a wider range of sample amount.

For the external standard GC/MS method, material that was chloroform extracted and methanol precipitated was used as the standard. For the internal standard GC/MS method, the area and amount of the standard (benzoic acid) was used with the sum of the areas from methyl-3-hydroxy alkanoates from known amounts of purified PHAs to develop a response factor. When the two GC/MS methods of calculation are compared between the three strains and the different carbon sources, for the most part, the results using the external standard (%PHA (GC-Ext)) and the internal standard (%PHA (GC-Int)) are close (33% external vs 34% internal) (Tables 3–5). Using the external standard method, strains B-14875, KT2440, and GN112 averaged 29% (± 4.5) PHA, 34% (± 5.7) PHA, and 35% (± 4.5) PHA, respectively. Using the internal standard method, strains B-14875, KT2440, and GN112 averaged 32% (± 4.8) PHA, 34% (± 3.7) PHA, and 36% (± 4.4) PHA, respectively. Although the results using the external and internal standards are quite similar in this instance, it should be noted that the reference factor for the internal standard was an average of values derived from a wide range of sample amounts (S1 File). Because there is a wide variation in response factor values for the internal standard, there is a greater risk of calculating an inaccurate internal standard response factor especially if a laboratory were to use only a single amount of sample for its calculations.

For the gravimetric method, lyophilized cells were extracted with warm chloroform, and the extract and washings were filtered through a glass fiber filter. The extracts were then evaporated overnight and the remaining material was weighed (%PHA Init). The evaporated material was then redissolved in chloroform and precipitated overnight in cold methanol (%PHA MeOH). From the data presented in Tables 3–5, the initial chloroform extract for strains B-14875, KT2440, and GN112 averaged 41% PHA (± 5.9), 43% (± 3.8) PHA, and 42% (± 4.3) PHA, respectively. For the methanol precipitated material, the levels for strains B-14875, KT2440, and GN112 averaged 23% PHA (± 5.8), 30% (± 6.1) PHA, and 30% (± 5.5) PHA, respectively. That the initial extract percentage (%PHA Init) was usually greater than that determined by the GC/MS method suggests that the initial extraction includes non-PHA chloroform soluble material (most likely membrane lipids and free fatty acids). With respect to the difference between the percent PHA in the initial extract (%PHA Init) and the percent PHA in the methanol precipitated PHA (%PHA MeOH), this can be explained by assuming that small molecular weight PHA and other chloroform soluble lipids in the initial extract would not be precipitated in cold methanol. Both GC methods were correlated with the gravimetric determination; though the strength of the correlations was not high, the GC-external method was more correlated with the final gravimetric values than the internal standard method (GC_ext_ r^2^ = 0.63, p < 2.2E-16; GC_int_ r^2^ = 0.34, p = 3.4E-5). These results are consistent with those observed by Riis and Mai (1988) although they examined the homopolymer polyhydroxybutyrate produced in *Methylobacterium* sp. [51].

### Characterization of PHA from *P*. *putida* strains B-14875, KT2440, and GN112

Table 6 shows the mole percent composition of the PHA present in the dried cells of each strain. With all three strains, 3-hydroxyheptanoate was the dominant monomer when grown on heptanoate, and 3-hydroxyoctanoate was the dominant monomer when grown on octanoate. When cells were grown on higher molecular weight, even-numbered alkanoates (C10 through C14), 3-hydroxyoctanoate was the dominate monomer (about 50%). 3-hydroxydecanoate was present at about 35%, and 3-hydroxyhexanoate was typically present in 6–7%. In the same manner, when cells were grown on odd-numbered alkanoates (C9 through C13), 3-hydroxynonanoate was the dominant monomer (50% to 70%) with 3-hydroxyheptanoate present at 18% to 28%. When the cells were grown on heptanoate through nonanoate, a small amount of the 2-carbon-larger 3-hydroxyalkanoates were produced. When cells were grown on decanoate through myristate, the corresponding 3-hydroxyalkanoate was the largest monomer in the produced PHA. However, as the size of the fed alkanoate increased, the amount of the corresponding 3-hydroxyalkanoate decreased, i.e. from about 40% on decanoate to about 1% on myristate. Also notable is that when cells were grown on substrates ranging from decanoate to myristate, either 3-hydroxyoctanoate (for even numbered alkanoates) or 3-hydroxynonanoate (for odd numbered alkanoates) is the dominant monomer in the resulting PHA. Similar results have been previously obtained [12–14, 52] with respect to growth on heptanoate and octanoate as well as on longer chain alkanoates.

The foregoing suggests that the PHA synthases may prefer 3-hydroxyheptanoyl-, 3-hydroxyoctanoyl-, or 3-hydroxynonanoyl-coenzyme A. Alternatively, heptanoate-, octanoate-, and nonanoate-CoA could be the preferred substrate for phaJ, fadB (S-(+)-3-hydroxyacyl epimerase), fadBA (3-keto-acyl-CoA reductase), or any combination of these enzymes which are in the metabolic pathway for PHA biosynthesis [53].

In addition, the molecular weights of the extracted polymers from cells of strains B-14875 and KT2440 grown on the alkanoates were determined (S1 Table). In general, the molecular weights varied from 20,000 to 70,000 with a polydispersity of 1.2 to 1.6. These molecular weights are consistent with what others have observed [52, 54].

## Conclusion

This report examines the synthesis of mcl-PHAs in three strains of *P*. *putida* using a range of alkanoic acids as carbon sources from C7 to C14. Significant to this study is that the same concentration of carbon contents was supplied to the bacteria which allowed us to distinguish the different responses of the strains to longer chain length carbon sources. Some other studies that have compared different length carbon feedstocks used a constant percentage or constant molar amount of substrate which would obscure the differences detected in this work. This study, as have others, shows that PHAs produced on heptanoate and octanoate are composed of primarily 3-hydroxyheptanoate and 3-hydroxyoctanoate, respectively. With longer chain alkanoates as the carbon source, these strains produce copolymers that favor the incorporation of 3-hydroxyoctanoate (with an even-numbered alkanoate as the carbon source) or 3-hydroxynonanoate (with an odd-numbered alkanoate as the carbon source). In addition, we demonstrate the variation in apparent PHB titers depending on the quantification method employed. Also notable was the fact that the CDW produced under the conditions described for shake flask cultures in this work are significantly greater than that described for most other mineral salts-based media. Ultimately this is important because production processes will be based on defined salts media rather than complex media in order to reduce costs. Finally, the fact that the GN112 strain with numerous deleted genes had higher PHA titers highlights the potential for using strains with decreased metabolic loads in biopolymer production.

## Supporting information

S1 File Comparing internal standard reference factors to external standard method for GC/MS analysis. (DOCX)

S1 Table GPC determination of molecular weights of methanol precipitated PHA. (DOCX)
