# Correction: Polyhydroxyalkanoate production in Pseudomonas putida from alkanoic acids of varying lengths

**DOI:** 10.1371/journal.pone.0337084

**Published:** 2025-11-17

**Authors:** W. Dirk Sikkema, Andrew J. Cal, Upul I. Hathwaik, William J. Orts, Charles C. Lee

The captions for Table 3 is misplaced in the article. Please see the complete, correct Table 3 caption here.

**Table 3:** Growth and PHA production of *P. putida* B-14875.

Carbon		24 hrs	48 hrs	68 hrs	pH @	CDW	%PHA	%PHA	%PHA	%PHA
**Source**		**OD**_**600**_	**OD**_**600**_	**OD**_**600**_	**68 hrs**	**(g/L)**	**(GC-Ext)**	**(GC-Int)**	**Init**	**MeOH**
**Hep**	**Avg**	3.99	7.96	11.45	7.81	4.97	23.47	30.04	44.61	12.63
	**SD**	0.20	1.09	2.35	0.30	0.42	10.78	9.98	8.97	6.07
**Oct**	**Avg**	5.24	9.01	12.47	7.77	5.56	33.22	34.10	46.86	19.09
	**SD**	0.28	1.17	1.18	0.16	0.35	6.83	4.92	8.28	3.72
**Non**	**Avg**	4.31	7.00	12.92	7.18	4.05	28.78	33.35	40.20	28.36
	**SD**	0.47	1.52	3.81	0.52	0.37	10.71	7.83	4.77	4.87
**Dec**	**Avg**	3.55	6.39	10.75	6.83	4.43	27.30	31.32	36.74	26.79
	**SD**	1.34	0.46	1.17	0.30	0.56	4.10	4.43	2.96	2.10
**UnD**	**Avg**	4.96	7.67	11.62	6.64	4.72	26.41	38.49	37.98	20.70
	**SD**	0.17	0.22	3.10	0.38	0.99	4.99	5.78	3.28	0.49
**Lau**	**Avg**	5.11	8.91	10.65	6.84	4.56	32.17	30.19	44.98	30.78
	**SD**	0.27	0.81	2.24	0.34	0.67	4.64	3.72	5.87	4.06
**TrD**	**Avg**	6.14	10.60	14.18	7.60	6.12	34.64	35.25	44.06	21.99
	**SD**	0.35	0.73	1.22	1.17	0.28	8.05	6.60	5.42	5.11
**Myr**	**Avg**	2.89	7.81	13.87	7.11	5.07	22.66	22.41	29.03	21.05
	**SD**	0.05	0.50	1.33	0.22	0.24	4.07	3.94	5.74	4.50

“Hep”, “Oct”, “Non”, “Dec”, “UnD”, “Lau”, “TrD”, “Myr” are heptanoate, octanoate, nonanoate, decanoate, undecanoate, laurate, tridecanoate, and myristate, respectively, and correspond to the sodium salt of the alkanoic acid fed to each culture. Each number of “Avg” represents the average of six flasks. “SD” represents the standard deviation of the average above. “OD_600_” is the optical density of the culture at the time indicated. “CDW” corresponds to the cell dry weight after lyophilization of the collected cells. “(GC-Ext)” equals the percent PHA determined from the methanolysis of whole cells using purified PHA from a similar carbon source as an external standard. “(GC-Int)” equals the percent PHA determined from the methanolysis of whole cells using PHA from a similar carbon source as the standard relative to methyl benzoate as the internal standard. “Init” equals the initial percent mass of chloroform extracted material from freeze dried cells. “MeOH” equals the percent mass of the chloroform extracted, methanol precipitated material.
