# Supplementary material for: Polyhydroxyalkanoate production in Pseudomonas putida from alkanoic acids of varying lengths
Source: PLoS One. 2023 Jul 20;18(7):e0284377. doi: 10.1371/journal.pone.0284377 (PMC10358918; doi:10.1371/journal.pone.0284377)
Supplement: S1 File — (DOCX) [file pone.0284377.s001.docx]

**SUPPLEMENTAL MATERIALS**

Polyhydroxyalkanoate production in *Pseudomonas putida* from alkanoic acids of varying lengths

W. Dirk Sikkema, Andrew J. Cal, Upul I. Hathwaik, William J. Orts, and Charles C. Lee

**S1 File. Comparing internal standard reference factors to external standard method for GC/MS analysis.**

The typical GC/MS method of quantification uses benzoic acid as an internal standard. The below table uses a wide range of sample (octanoate) and a constant amount of benzoic acid. As can be seen, depending on the amount of sample, the response factor (RF) varies significantly with decreasing values as less sample is used. An average of the response factors was used for our analysis. However, if a study were to use an RF derived solely from a single amount of sample, the results would vary widely depending on the amount of sample used. Thus, it would be useful for studies to explicitly describe how their RF values were calculated when using an internal standard.

|  |  |  |  |  |  |
| --- | --- | --- | --- | --- | --- |
| Sample (mg) | Sample-area | Benzoic acid (mg) | Benzoic acid-area |  | RF |
| 4.58 | 7154552 | 2.252 | 6354157 |  | 1.8062 |
| 9.16 | 10978020 | 2.252 | 6497496 |  | 2.4074 |
| 13.74 | 15840322 | 2.252 | 6417728 |  | 2.4719 |
| 18.32 | 19438553 | 2.252 | 6497886 |  | 2.7194 |
| 22.90 | 23158328 | 2.252 | 5960926 |  | 2.6174 |
| 4.62 | 6458230 | 2.252 | 6321033 |  | 2.0062 |
| 9.23 | 11042603 | 2.252 | 6145054 |  | 2.2813 |
| 13.85 | 15514272 | 2.252 | 6408080 |  | 2.5399 |
| 18.46 | 19222628 | 2.252 | 6479172 |  | 2.7635 |
| 23.08 | 21475914 | 2.252 | 6571086 |  | 3.1358 |
| 4.54 | 6962706 | 2.252 | 7129930 |  | 2.0626 |
| 9.07 | 11442910 | 2.252 | 6736394 |  | 2.3715 |
| 13.61 | 16565477 | 2.252 | 7486021 |  | 2.7307 |
| 18.14 | 21487738 | 2.252 | 7612642 |  | 2.8544 |
| 22.68 | 25090213 | 2.252 | 7847704 |  | 3.1500 |
| 4.87 | 8360168 | 2.252 | 7098147 |  | 1.8368 |
| 9.74 | 14074853 | 2.252 | 7417679 |  | 2.2803 |
| 14.62 | 18018698 | 2.252 | 7269683 |  | 2.6185 |
| 19.49 | 21418958 | 2.252 | 7326971 |  | 2.9602 |
| 24.36 | 30644821 | 2.252 | 7235733 |  | 2.5541 |
|  |  |  |  |  |  |
|  |  |  |  | Mean | 2.5084 |
|  |  |  |  | SD | 0.39 |
|  |  |  |  | CV | 15.42 |

In contrast, using an external standard is fairly linear over a range of PHA amounts (5 – 25 mg) as can be seen in the graph below.
