# Supplementary material for: Polyhydroxyalkanoate production in Pseudomonas putida from alkanoic acids of varying lengths
Source: PLoS One. 2023 Jul 20;18(7):e0284377. doi: 10.1371/journal.pone.0284377 (PMC10358918; doi:10.1371/journal.pone.0284377)
Supplement: S1 Table — (DOCX) [file pone.0284377.s002.docx]

**SUPPLEMENTAL MATERIALS**

Polyhydroxyalkanoate production in *Pseudomonas putida* from alkanoic acids of varying lengths

W. Dirk Sikkema, Andrew J. Cal, Upul I. Hathwaik, William J. Orts, and Charles C. Lee

**S1 Table. GPC determination of molecular weights of methanol precipitated PHA.**

|  |  |  |  |
| --- | --- | --- | --- |
|  |  |  | **Dispersity** |
| **Strain** | **Mn (kDa)** | **Mw (kDa)** | **(Mw/Mn)** |
| B-14875  Hep | 70.2 | 84.5 | 1.2 |
| Dec | 37.2 | 43.1 | 1.2 |
| UnD | 24.0 | 38.3 | 1.6 |
| Lau | 20.4 | 32.2 | 1.6 |
| TrD | 26.2 | 40.3 | 1.5 |
| Myr | 24.0 | 35.9 | 1.5 |
|  |  |  |  |
| KT2440 |  |  |  |
| Hep | 40.2 | 63.6 | 1.6 |
| Oct | 32.8 | 52.4 | 1.6 |
| Non | 37.9 | 52.9 | 1.5 |
| Dec | 30.1 | 47.2 | 1.6 |
| UnD | 30.8 | 48.6 | 1.6 |
| Lau | 30.7 | 47.7 | 1.6 |
| TrD | 48.4 | 55.7 | 1.2 |
| Myr | 45.3 | 51.7 | 1.1 |

“Hep”, “Oct”, “Non”, “Dec”, “UnD”, “Lau”, “TrD”, “Myr” are heptanoate, octanoate, nonanoate, decanoate, undecanoate, laurate, tridecanoate, and myristate, respectively, and correspond to the sodium salt of the alkanoic acid fed to each culture.
